# Skillful Use of Technologies of the Extended Mind Illuminate Practical Paths Toward an Ethics of Consciousness

**DOI:** 10.3389/fpsyg.2018.01251

**Published:** 2018-07-19

**Authors:** Saskia K. Nagel, Peter B. Reiner

**Affiliations:** ^1^Human Technology Center, RWTH Aachen University, Aachen, Germany; ^2^Department of Psychiatry, University of British Columbia, Vancouver, BC, Canada

**Keywords:** technologies of the extended mind, neuroethics, mind, consciousness ethics, technologically embedded

A primary concern of the field of neuroethics has been the sanctity of our minds. Issues such as the privacy of thought, worries about manipulating emotions or memories and most prominent of all, concerns about intervening to enhance our brain's abilities beyond species-typical functioning have dominated this young discipline for the past decade. One issue whose consideration has been notably absent relates to which states of consciousness we would like to—or even should—pursue. This has been highlighted by Thomas Metzinger who suggests that we might profit from developing a new field of applied ethics to address this issue: consciousness ethics (Metzinger, 2009). Such a field would be explicitly concerned with questions about what might constitute a desirable state of consciousness and then explore avenues toward achieving those goals.

Metzinger's treatment is not exhaustive, but he does suggest that a desirable state consciousness would satisfy at least three conditions: (1) minimizing suffering of all beings capable of suffering; (2) possessing epistemic potential; (3) increasing the probability of occurrences of valuable types of experiences. With this outline in hand, we can begin to explore the question of how we might achieve those goals. In considering this issue, we emphasize approaches that are realistic today. Thus, hypotheticals about drugs that allow us to become morally superior beings or that provide “limitless” cognitive abilities are theoretically interesting, but not practically useful today. We suggest that a particularly useful starting point for such a discourse is to consider the immense impact that our ever-present algorithmic devices have on our conscious states, for these technologies have already become potent actors in our everyday conscious experience. Indeed, it is increasingly well-recognized that modern-day humans have become deeply technologically embedded beings, most notably for having extensively blended our biological brains with our algorithmic devices. By considering the impact of our technological environment upon our mental states, we find that these devices represent real-world manifestations of the extended mind thesis (Clark and Chalmers, 1998). This development, as Neil Levy so presciently noted, serves to dramatically *expand* the scope of neuroethics rather than detracting from its importance (Levy, 2007). To emphasize this relationship, we call these devices Technologies of the Extended Mind (TEMs) (Fitz and Reiner, 2016; Nagel et al., 2016)—devices that represent parts of our minds that reside outside of our biological brains. Recognizing this forces us to reflect about what it means to be a human being navigating the modern world (Reiner and Nagel, 2017), and serves as an illuminating example for considering an ethics of consciousness.

We have suggested that not every algorithmic function carried out by a device external to the brain qualifies them as a TEM (Reiner and Nagel, 2017). Rather, devices that qualify are those in which there is a relatively seamless interaction between brain and algorithm such that we *perceive* the algorithm as being a *bona fide* extension of our minds. Users need not explicitly reflect and label the algorithm as a TEM, but if their praxis is consistent with this provisional definition, it should qualify. If we accept the notion of TEMs as extensions of our minds, the key question that an ethics of consciousness raises is, what *should* our minds become when they are composed of a blend of brain and external resources? There is no easy answer to this question, but one thing that we can conclude readily is that we should be careful about who designs the extensions of our minds.

Using the smartphone as a canonical example, the contemporary situation looks something like this: our biological brains pick and choose from the vast assortment of algorithmic apps available to us. Some of these may rise to the level of TEMs, becoming seamlessly a part of the cognitive resources that we use whilst navigating the world around us. A key question, informed by applying a virtue ethics lens to technology (such as explored by Vallor, 2016), might ask how skillfully we are engaging with this choice. Do we stop and consider what kind of mind we will have if we include this or the other app as part of our minds? Do we heed Metzinger's advice and reflect upon whether the app minimizes suffering, has epistemic potential, or increases the likelihood of valuable experiences?

This analysis can be further developed to think about *how* a particular app helps to achieve these objectives. Does the app take over the process of minimizing suffering, freeing people from considering such matters – for example by automatically contributing to charity, or does it train our biological brains to do so, as a mindfulness app might? Does the app itself have epistemic potential, or does its use result in deeper insight on our part? Does the app increase the likelihood that we will have valuable experiences in life? Does having an app that serves as a TEM, in and of itself, provide us with more valuable experiences?

We suggest that a further dimension might be worthy of consideration: the consequences of using a particular app, in particular the degree to which the app supplants or enhances the function of our biological brains. While the concept of extended mind that we subscribe to can integrate brain, smartphone, and more, in the context we discuss here, sharply distinguishing between brain and smartphone provides clarity. At one end of the spectrum, an app may act to instrumentally achieve a particular objective, but may also degrade our brains' abilities to carry out similar tasks. For example, many people use the GPS mapping function on their smartphones to navigate from one place to another, and some scholars have suggested that regular use degrades our brains' navigational skills (McKinlay, 2016). A similar situation obtains with the ready availability of calculators, originally as a stand-alone device but now included as an app on every smartphone. People who heavily rely on calculators struggle with the (learned) ability to carry out mathematical problems either in their heads or using pencil and paper (Miles, 2008). At the other end of the spectrum are apps that help our brains learn how to better manage our lives so that objectives that are meaningful to us are attained. A cardinal example would be any of the many popular meditation apps that are purported to help people achieve greater mindfulness. By training one's mind to remain focused on the task at hand (for example, breathing), the app helps people achieve an instrumentally valuable objective by strengthening the brain's ability to retain focus rather than having the app take over the task.

These seem like weighty issues to consider when simply adding an app to our smartphone. But, in select instances, that is not what we are doing; rather, we are adding an algorithmic agent to our extended minds. Under such conditions, the value of asking these questions changes considerably.

In many ways, these considerations suggest that we need to exercise wisdom when accepting an algorithmic agent as an extension of our minds. Adults should, in theory, have the requisite intellectual resources to do so, but in practice the vast majority of people do not consider these issues when adding apps to their smartphones. This is surely a skill that can and should be developed by the populace at large. Even more problematic is the situation with adolescents and younger children who often have the ability to add apps but are not likely to have sufficiently mature cognitive resources to address these issues in a manner that is consistent with their long-term best interests. Finding ways to impart such wisdom practices to our relationship with our devices is an important objective toward developing an ethics of consciousness that includes our algorithmic companions.

## Author contributions

All authors listed have made a substantial, direct and intellectual contribution to the work, and approved it for publication.

### Conflict of interest statement

The authors declare that the research was conducted in the absence of any commercial or financial relationships that could be construed as a potential conflict of interest.
